# Constitutive, but Not Challenge-Induced, Interleukin-10 Production Is Robust in Acute Pre-Pubescent Protein and Energy Deficits: New Support for the Tolerance Hypothesis of Malnutrition-Associated Immune Depression Based on Cytokine Production *in vivo*

**DOI:** 10.3390/ijerph8010117

**Published:** 2011-01-13

**Authors:** Jennifer M. Monk, Tessa A.M. Steevels, Lyn M. Hillyer, Bill Woodward

**Affiliations:** Department of Human Health and Nutritional Sciences, University of Guelph, Guelph, Ontario, N1G 2W1, Canada; E-Mails: jmonk@uoguelph.ca (J.M.M.); T.A.M.Steevels@umcutrecht.nl (T.A.M.S.); lhillyer@uoguelph.ca (L.M.H.)

**Keywords:** anti-inflammatory, immune tolerance, interleukin-10, mouse, protein-energy malnutrition

## Abstract

The tolerance model of acute (*i.e.*, wasting) pre-pubescent protein and energy deficits proposes that the immune depression characteristic of these pathologies reflects an intact anti-inflammatory form of immune competence that reduces the risk of autoimmune reactions to catabolically released self antigens. A cornerstone of this proposition is the finding that constitutive (first-tier) interleukin(IL)-10 production is sustained even into the advanced stages of acute malnutrition. The IL-10 response to inflammatory challenge constitutes a second tier of anti-inflammatory regulation and was the focus of this investigation. Weanling mice consumed a complete diet ad libitum, a low-protein diet ad libitum (mimicking incipient kwashiorkor), or the complete diet in restricted daily quantities (mimicking marasmus), and their second-tier IL-10 production was determined both *in vitro* and *in vivo* using lipopolysaccharide (LPS) and anti-CD3 as stimulants of innate and adaptive defences, respectively. Both early (3 days) and advanced (14 days) stages of wasting pathology were examined and three main outcomes emerged. First, classic *in vitro* systems are unreliable for discerning cytokine production *in vivo*. Secondly, in diverse forms of acute malnutrition declining challenge-induced IL-10 production may provide an early sign that anti-inflammatory control over immune competence is failing. Thirdly, and most fundamentally, the investigation provides new support for the tolerance model of malnutrition-associated inflammatory immune depression.

## 1. Introduction

In its most severe forms, acute pre-pubescent malnutrition manifests in two distinct pathologies, namely marasmus and kwashiorkor, and susceptibility to opportunistic infections is characteristic of both [1,2]. Depressed inflammatory immune competence is widely accepted as the link between malnutrition and susceptibility to infection [1,2], and proof-of-principle evidence demonstrates that inflammatory capabilities can be increased independently of ongoing weight loss in acute pre-pubescent deficits of protein and energy [3,4]. There is reason for optimism, therefore, that improved understanding of malnutrition-associated immunological change will lead to improved management of infection even in the most advanced stages of acute protein and energy deficits.

Malnutrition-associated immune depression is widely attributed to a disintegrative loss of inflammatory capacities [5–7] and the loss is tacitly assumed to include the ability to both generate and resolve an inflammatory response. However this longstanding paradigm is under challenge by a proposition centered on regulated preservation of anti-inflammatory immune capability [1,5–7]. Briefly, the so-called “tolerance model” identifies malnutrition-related immune depression as a physiological strategy to reduce the risk of inflammatory autoimmune reactions in the face of catabolically-released self antigens [3,7]. The model acknowledges the resulting cost in terms of susceptibility to infection [7] and accommodates the biologically trivial endpoint that a disintegrative loss of all forms of immune competence must ultimately prevail if wasting disease is permitted to proceed indefinitely.

The tolerance model centers on three anti-inflammatory mediators, namely the glucocorticoids, transforming growth factor(TGF)-β and interleukin(IL)-10. This hormonal triad plays a determining role in physiological anti-inflammatory control and in the maintenance of self tolerance [8–13]. The constitutive blood levels of these mediators are positioned, collectively, to respond when an inflammatory challenge is first encountered and, consequently, they represent the first tier of anti-inflammatory and regulatory immune competence. Recently, high constitutive blood levels of each member of this anti-inflammatory triad have been reported in weanling mouse models of marasmus and incipient kwashiorkor [5–7,14], models which consistently reproduce the critical features of these pediatric pathologies [5,14–18]. Further, remarkably, the systemic rate of constitutive IL-10 production is at least sustained, and can even be elevated, into the advanced stages of wasting pathology in the same high-fidelity mouse models of pre-pubescent malnutrition [7], thereby providing the first mechanistic evidence supporting the tolerance model.

In addition to their constitutive production, anti-inflammatory mediators are released as a component of any competent response to inflammatory stimuli [19]. Thus, stimulus-dependent production of soluble mediators may be regarded as a second tier of anti-inflammatory and tolerogenic immune regulation. The concept of layers of anti-inflammatory regulation has been espoused previously and both innate and adaptive types of second-tier responses can be identified [20]. IL-10 is a particularly important anti-inflammatory mediator that regulates both innate and adaptive immune responses [21,22]. However, information is unavailable pertaining to the influence of acute pre-pubescent protein and energy deficits on second-tier IL-10 production. Therefore, the objective of this investigation was to assess the influence of metabolically distinct forms of acute malnutrition on innate and adaptive-type second-tier IL-10 production using weanling mouse models known to sustain constitutive production of this anti-inflammatory cytokine. Within this objective, the primary purpose was to assess stimulant-induced cytokine production *in vivo*, whereas a secondary purpose was to determine whether the more common strategy of assessing the response to stimulation *in vitro* would yield a similar outcome. Lipopolysaccharide (LPS) potently activates innate immune defences through the toll-like receptor(TLR)-4 [23] and was selected to elicit an innate-type response. The adaptive-type response was investigated by stimulating the effector/memory T cell compartment, an important source of IL-10 in the context of acquired immune competence [22], using anti-CD3 as a polyclonal stimulant.

## 2. Materials and Methods

### 2.1. Animals and Housing Facilities

Male and female C57BL/6J mice were obtained from an in-house breeding colony derived from animals originally purchased from the Jackson Laboratory (Bar Harbor, ME). Caging and housing conditions were exactly as described previously [5–7,14–18]. Briefly, the animals were held in a windowless room supplied with fluorescent lighting on a 14L:10D schedule, while temperature and relative humidity were regulated at 25–26 °C and 60–70%, respectively. This investigation was approved by the Animal Care Committee of the University of Guelph in accordance with the Canadian Council on Animal Care.

### 2.2. Diets

Eighteen-day-old animals were weaned and acclimated overnight to a complete purified diet described in detail elsewhere [24]. The diet was made using spray-dried egg white as its source of nitrogen together with cornstarch, glucose, corn oil, cellulose and a micronutrient supplement formulated according to the classic AIN-76 diet for rodents. A typical proximate analysis of the diet (as-fed basis) is 93% dry matter, 18% crude protein, 8% ether extract, 2.6% ash, 3% crude fibre and 17 kJ/g gross energy. Following the acclimation period, equal numbers of male and female 19-day-old mice were randomly assigned to one of three experimental groups, namely an age-matched control group consuming the complete purified diet ad libitum, a restricted-intake group consuming the complete purified diet in restricted daily quantities based on calculations that relate ad libitum food intake (g food/g body weight) to chronological age in the weanling mouse [15], or a low-protein group consuming ad libitum a nitrogen-deficient purified diet that was isocaloric with the complete formulation [5,16]. To make the low-protein diet, the complete diet was re-designed by weight-for-weight replacement of a portion of the cornstarch to achieve a crude protein level of 0.6%. The restricted-intake protocol produces a pathology that reproduces the critical features of pediatric marasmus, whereas the low-protein model closely resembles incipient pediatric kwashiorkor [5,14–18].

### 2.3. Experimental Design

Innate and adaptive capacities to produce IL-10 in response to appropriate stimulation were each assessed by means of two experiments, one conducted *in vivo* and the other *in vitro*. In all four experiments, cohorts of animals were maintained on each of the three dietary regimens for 14 days, *i.e.*, from 19 through 33 days of age. Additionally, in the two experiments centered on the *in vivo* response, a cohort of animals from each dietary protocol was examined after only 3 days (*i.e.*, at 22 days of age), a time point taken to represent early-stage weight loss in the malnourished groups [6,7,14]. Carcasses from animals of all experiments were stored at −20 °C to await analysis. It should be noted that female estrous cycling (at least on the part of age-matched control animals) may have contributed variance to the design of the studies involving a 14-day feeding period. Part of the intent, however, was to determine whether malnutrition-related influences could be discerned against a background of normal physiological variability.

IL-10 production *in vivo* in response to stimulation by either LPS or anti-CD3 was assessed at day 3 (early time point) and day 14 (advanced weight loss), thus yielding a 2 × 2 design in which the statistical main effects were diet and stage of weight loss. Eight mice (four males and four females) were included within each dietary group at each time point. Unstimulated negative controls, effectively providing a measure of constitutive IL-10 production, were included within each dietary group and stage of weight loss in both experiments (n = 16, eight males and eight females per group, in the investigation of the innate response to LPS and n = 3, two males and one female per group, in the study of the adaptive T cell response).

A sample size of six per dietary group was used to investigate the innate response to LPS *in vitro*, whereas the *in vitro* response of effector/memory T cells (to stimulation by anti-CD3) was investigated using a sample of ten mice in the age-matched control group and eight animals in each of the malnourished groups. Equal numbers of males and females were included in all experimental groups. In order to obtain sufficient numbers of cells from secondary lymphoid organs (spleen combined with mesenteric and inguinal lymph nodes), pooled samples were required for mice subjected to the low-protein and restricted-intake groups. Thus, in the study of the *in vitro* response to LPS each sample from the low-protein and restricted-intake groups comprised two and four mice, respectively, whereas 2–4 low-protein mice and 2–8 restricted-intake mice were pooled to produce each sample of malnourished animals in the study of the *in vitro* response of effector/memory T cells. Each pooled sample included equal numbers of males and females and constituted a single degree of freedom for the purpose of statistical analysis. The experiments centered on *in vitro* responses were conducted according to a 2 × 2 design with diet and *in vitro* stimulation as the statistical main effects, and cells were harvested for study at the end of the 14-day experimental period.

### 2.4. *In vivo* Stimulation of IL-10 and Assessment of Cytokine Production by *in vivo* Capture Assay

An *in vivo* cytokine capture assay kit for murine IL-10 (BD Biosciences, Mississauga, Canada, catalogue # 558072) was utilized as described previously [7]. Briefly, each animal received an aseptic intraperitoneal injection of 10 μg of biotin-conjugated anti-mouse IL-10 (“capture”) antibody delivered in 200 μL of endotoxin-free physiological saline (Vétoquinol N.–A. Inc., Lavaltrie, Québec, Canada; henceforth, “saline”). Animals used for assessment of the innate response to endotoxin received a simultaneous intraperitoneal injection of LPS (Escherichia coli 055:B5, Sigma-Aldrich, St. Louis, USA), 1 μg/g body weight, whereas mice used in the investigation of T cell-derived IL-10 were given a simultaneous intraperitoneal injection of 10 μg of anti-mouse CD3 (clone: 145-2C11, Armenian hamster IgG; BD Bioscience, San Diego, CA, USA). Negative controls for the mice given LPS each received 10 μg of the capture antibody, only, but negative controls for the mice stimulated with anti-CD3 received 10 μg of capture antibody plus 10 μg of Armenian hamster IgG (catalog # G235-2356, BD Biosciences, San Diego, CA, USA) by intraperitoneal injection in 200 μL of sterile saline. Blood samples were collected 4 hours later to permit analysis of the concentration of IL-10 that had accumulated in molecular complexes with the capture antibody. LPS and anti-CD3 have previously been utilized successfully in combination with the capture assay [25,26]. Moreover, four hours provides ample time to generate elevated serum IL-10 concentrations in response to stimulation either by LPS [25,26] or by anti-CD3 [26].

A sandwich ELISA was used to detect the antibody/cytokine complexes and was performed with reagents provided in the capture assay kit (BD Biosciences). The capture reagent of the ELISA was a rat monoclonal antibody that recognized a different IL-10 epitope than the biotin-conjugated capture antibody used *in vivo*, and the detection reagent was streptavidin conjugated with horse radish peroxidase. In a minor departure from the manufacturer’s instructions, an additional blocking step was included using 10% fetal bovine serum (Sigma Chemical) in sterile PBS (1.0 mM, pH 7.3) following the capture stage of the ELISA. Outcomes were quantified by optical density using a Vmax kinetic plate reader (Molecular Devices Corp., Menlo Park, CA) set for absorbance at 450 nm with wavelength correction based on absorbance at 570 nm.

The reliability (intra-assay coefficient of variation) and detection limit of each assay were estimated as described previously [27] and averaged 4.3% and 0.14 pg/mL (of IL-10 in molecular complex) respectively. The standard curves for the assay were linear (mean R^2^ = 0.99) over a concentration range up to 2,000 pg/mL. All samples fell within the linear portion of the curve and exceeded the detection limit of the assay.

### 2.5. Blood Sampling Procedure for the *in vivo* Capture Assay

As described previously [14] blood samples were collected from the orbital plexus while the mice were under CO_2_ anesthesia, and the mice were then killed by cervical dislocation without recovering consciousness. Blood was allowed to clot at room temperature for 30–45 minutes and the resulting serum was stored at −80 °C to await analysis.

### 2.6. Procedure to Obtain Mononuclear Cell Suspensions for *in vitro* Stimulation

After measurement of body weight, mice were anesthetized with CO_2_ and killed by cervical dislocation without recovering consciousness. The spleen and the mesenteric and inguinal lymph nodes were removed aseptically, diced together and forced through a sterile stainless steel wire screen (100-mesh) into RPMI 1640 medium (Flow Laboratories, Mississauga, Canada) containing 10% heat-inactivated fetal bovine serum (Sigma Chemical, St. Louis, MO), 1 mmol/L HEPES (ICN Biomedicals, Aurora, OH), 10^5^ U/L penicillin and 100 mg/L streptomycin (hereafter designated “complete medium”). A single-cell suspension of mononuclear cells was produced by discontinuous gradient centrifugation as described previously [28]. Cell numbers were determined using a hemocytometer and viability, assessed by eosin Y exclusion, always exceeded 95%.

### 2.7. *In vitro* Stimulation of Mononuclear Cells with LPS to Elicit IL-10 Production

Each well of a 96-well V-bottom plate (catalog #249662, Nalge Nunc International), received 2 × 10^5^ viable mononuclear cells in 190 μL complete medium. Subsequently, 10 μL of complete medium was added to half of the culture wells to generate unstimulated negative control cultures while the remaining wells received 10 μL of LPS (Escherichia coli 055:B5, Sigma–Aldrich, St. Louis, USA) diluted in complete medium to achieve a final concentration of 10 μg/mL in each well. All cultures were run in triplicate and incubated at 37 °C for 24 h. After incubation, supernatants from each set of triplicate wells were produced by centrifugation at 200 g and the supernatants were pooled, subdivided into aliquots and stored at −80 °C.

### 2.8. *In vitro* Stimulation of T Cells with Anti-CD3 to Elicit IL-10 Production

Falcon plates (flat-bottom wells, Becton Dickinson Labware, New Jersey, USA, catalog #3072) were coated overnight at 4 °C with 200 μL 0.01 M phosphate-buffered saline (PBS, pH 7.3) containing 5 μg/mL anti-CD3 (clone: 145-2C11; Cedarlane Laboratories, Hornby, Canada). After coating, plates were washed twice with PBS and each well received 2 × 10^5^ viable mononuclear cells in 190 μL complete medium. Subsequently, 10 μL of PBS was added to half of the culture wells while the remaining test cultures received 10 μL of PBS containing anti-CD28 (clone: 37.51.1; Cedarlane Laboratories, Hornby, Canada) to achieve a final concentration of 20 μg/mL. Negative control cultures were produced using wells not coated with anti-CD3 and the cells were cultured in fluids comprising 190 μL complete medium together with 10 μL PBS. All cultures were incubated at 37 °C for 24 h. After incubation, plates were centrifuged for 1 min at 200 g, and supernatants from cultures representing the same dietary group and stimulus were pooled and stored at −80 °C.

### 2.9. Assay of IL-10 Concentrations Generated *in vitro*

A sandwich ELISA kit for assay of mouse IL-10 (BD Biosciences, Mississauga, ON, catalogue #555252) was applied to samples of culture fluids as described by the manufacturer. Outcomes based on triplicate assays were quantified by optical density using a Vmax kinetic plate reader (Molecular Devices Corp., Menlo Park, CA) set for absorbance at 450 nm with wavelength correction based on absorbance at 570 nm. Only the linear portions of standard curves were used (mean R^2^ = 0.97, at minimum), and all samples fell within this part of the curve. In the studies of innate and adaptive responses, the reliability of the assay (intra-assay coefficient of variation) averaged 5.8% and 8.1%, respectively, and its detection limit, determined as described elsewhere [27], averaged 2.2 and 1.2 pg/mL, respectively.

### 2.10. Assessment of Percentage CD3^+^ Cells in Mononuclear Cell Suspensions from Spleen and Lymph Nodes

Analyses were performed using a Becton-Dickinson FACSCalibur flow cytometer equipped with BD CellQuest TM software (2001). Generic aspects of staining procedures in this laboratory, including Fc receptor blockade, are described elsewhere [15,28]. Cells were subjected to single-color analysis by means of a phycoerythrin-conjugated anti-mouse CD3ɛ monoclonal antibody (clone: 145-2C11, Armenian hamster IgG, eBiosciences, San Diego, CA, USA) at a concentration of 0.2 μg per 250 × 10^3^ viable cells. Negative control samples were stained with biotin-conjugated hamster IgG (Cedarlane Laboratories, Hornby, ON) followed by phycoerythrin-conjugated streptavidin (Cedarlane Laboratories, Hornby, ON), respectively at concentrations of 0.2 μg and 0.15 μg per 250 × 10^3^ viable cells. Cells were incubated with staining reagents in the dark on ice for 40 min and then were fixed with paraformaldehyde (20 g/L) and analyzed immediately. Viability before staining was determined by eosin Y exclusion and always exceeded 95%. Each analysis was based on at least 10^4^ events after dead cells and residual erythrocytes were eliminated by gating on the basis of forward-angle light scatter.

### 2.11. Assessment of Carcass Composition

Carcasses from animals of both experiments were stored at −20 °C to await analysis. Analyses of dry matter and total lipid content were performed as described previously [14,15].

### 2.12. Statistical Analyses

Statistical analyses were conducted using the SAS system (SAS Institute, Cary, NC, USA) for Windows (version 9.0), and a predetermined upper limit of probability of P ≤ 0.05 was applied for statistical significance. Data were subjected to a two-way ANOVA followed, if justified by the level of statistical probability (P ≤ 0.05), by Tukey’s Studentized Range test. If a data set failed to exhibit normal distribution according to each of the four tests applied by the SAS program (P ≤ 0.05) then a transformation was utilized to bring it into conformity with this basic assumption of parametric testing.

## 3. Results

### 3.1. Distinct Weight Loss Pathologies Were Elicited by the Malnutrition Protocols

Growth indices for the experiments pertaining to the primary interest of this investigation, *i.e.*, IL-10 production *in vivo*, are shown in Table 1 (innate response to LPS) and Table 2 (T cell response to anti-CD3), and the results were comparable in the two experiments. Initial body weights did not differ among the dietary groups and body weight gain, food intake and carcass composition of the age-matched control group were comparable to previous results pertaining to C57BL/6J weanlings given free access to the complete diet used in this investigation at both time points [5,7,14,16,17,24]. The final body weights of the malnourished groups were lower than those of their corresponding age-matched control groups but did not differ from each other. In addition, the malnourished groups exhibited decreased food intakes relative to their corresponding age-matched controls, as seen previously [7,14–16]. Moreover, the malnourished groups exhibited a lower percentage of carcass fat than controls, but the extent of carcass fat loss was more pronounced in the restricted-intake group at both stages of weight loss that were assessed. Therefore, as reported elsewhere [5,17,29], the restricted-intake protocol induced a greater decrement in carcass energy than the low-protein protocol, and this was apparent even at an early stage of weight loss. Importantly, the wasting disease produced by the two malnutrition protocols was comparable to that reported in previous studies wherein inflammatory immune competence was shown to be depressed [15,30,31]. Similar outcomes with respect to growth indices were apparent in the experiments centered on the *in vitro* response to stimulation with LPS and anti-CD3 (results not shown).

### 3.2. IL-10 Production *in vivo* and *in vitro* in Response to Either LPS or Anti-CD3

#### 3.2.1. IL-10 capture *in vivo* following stimulation with LPS

Analysis of the data from the age-matched control groups (days 3 and 14) verified the LPS stimulation protocol for the purpose of this investigation. A two-way ANOVA revealed an IL-10 response to LPS (P = 0.002) that was dependent on chronological age (P = 0.003 for interaction between stimulus and age), and subsequent Least Squares Means comparisons revealed an LPS-induced increase in the blood concentration of captured IL-10 at 33 days of age (*i.e.*, day 14; means: 218 *vs.* 916 pg/mL, P = 0.0001) but no response to LPS at 22 days of age (*i.e.*, day 3; means: 408 *vs.* 422 pg/mL, P = 0.914). Thus, the LPS stimulus elicited IL-10 production in healthy pre-pubescent mice permitted to develop this response capability as a part of their normal ontogeny.

**Figure 1:**
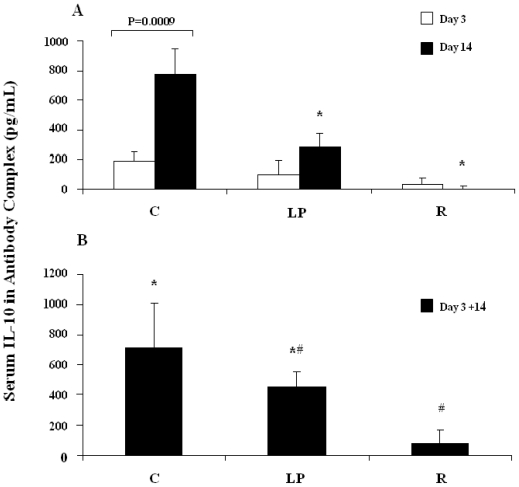
Serum concentration of IL-10 induced by LPS challenge (panel A) and anti-CD3 challenge (panel B) *in vivo* and complexed with biotin-conjugated anti-IL10 antibody.

Within the unstimulated control animals of the full data set (all diet groups and both ages), there was no effect of diet (P = 0.46) or stage of weight loss (P = 0.92) on the concentration of antibody-captured serum IL-10. Consequently, the data were pooled to yield a correction factor of 253 pg/mL, effectively an estimate of constitutive IL-10 production *in vivo*, which was applied, by subtraction, to each LPS-stimulated animal. Figure 1A, therefore, pertains to LPS-stimulated IL-10 production, only.

As shown in Figure 1A, a two-way ANOVA (pooled SEM = 0.80) revealed main effects of diet (P = 0.0001) and stage of weight loss (P = 0.012) together with a statistically significant interaction term (diet X stage, P = 0.025). Bars represent mean values (squared means of square root transformed data) and an SEM is shown for each mean. According to Least Squares Means analysis a lower response to LPS was apparent in both malnourished groups (LP: low-protein group; R: restricted-intake group) relative to age-matched controls (group C) when weight loss was in its advanced stages, *i.e.*, at day 14 (P ≤ 0.05, bars marked with an asterisk). In fact, an LPS response was undetectable in the restricted-intake group at this stage. By contrast, no difference was apparent between either of the malnourished groups and the age-matched control group at the early stage of weight loss (day 3). Moreover, comparison between the age-matched control groups (day 3 *vs.* day 14) confirmed the ontogeny-associated rise in IL-10 production in response to LPS stimulation (P = 0.0009, Least Squares Means). Thus, the normal ontogenetic rise in LPS-stimulated IL-10 production was attenuated as malnutrition progressed in both forms of wasting pathology.

#### 3.2.2. IL-10 capture *in vivo* following stimulation with anti-CD3

Analysis of the data from the age-matched control groups (days 3 and 14) verified the anti-CD3 stimulation protocol for the purpose of this investigation. A two-way ANOVA revealed an IL-10 response to anti-CD3 (P = 0.030) but no effect of chronological age (P = 0.340) and no interaction between the statistical main effects (P = 0.969). Thus, the anti-CD3 stimulus elicited an increase in the blood concentration of captured IL-10 (means: 221 *vs.* 820 pg/mL) in healthy pre-pubescent mice at both stages of ontogeny examined herein.

No effect of diet (P = 0.28) or stage of weight loss (P = 0.38) was apparent among the isotype control groups, and their average serum level of antibody-captured IL-10 was 297 pg/mL. Therefore this correction factor, corresponding to constitutive IL-10 production *in vivo*, was applied (by subtraction) to each anti-CD3-stimulated animal to ascertain the magnitude of the systemic IL-10 response to polyclonal T cell stimulation, and the outcome is shown in Figure 1B.

As shown in Figure 1B, a two-way ANOVA (pooled SEM = 1.60) revealed a diet-related effect (P = 0.016) but no effect of the stage of weight loss (P = 0.70) and no interaction term (diet X stage, P = 0.26). Consequently, the data were combined across stages of weight loss to produce the results shown in the Figure. Bars represent mean values (squared means of square root transformed data) and an SEM is shown for each mean. Bars not sharing a symbol differ from each other (P ≤ 0.05) according to Tukey’s Studentized Range test. As revealed by the Figure, the 4-hour accumulation of captured IL-10 in the blood of the restricted-intake group (group R) was depressed, independently of the stage of weight loss, relative to age-matched controls (group C). Conversely, the response of the low-protein (LP) group to polyclonal T cell stimulation did not differ from that of either the age-matched controls or the restricted intake group.

#### 3.2.3. IL-10 production by mononuclear cells stimulated *in vitro* with LPS

**Figure 2:**
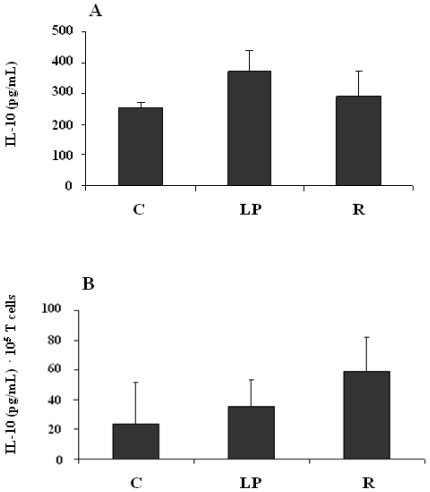
Concentration of IL-10 in cultures of mononuclear cells from spleen and lymph nodes in response to 24 hours of stimulation *in vitro* with either LPS (panel A) or anti-CD3 (panel B).

Data pertaining to the *in vitro* production of IL-10 in response to LPS were subjected to a two-way ANOVA (main effects being diet and stimulus, the latter discerned by comparing cultures with and without LPS). The outcome revealed an effect of stimulus (P < 0.0001) but no effect of diet (P = 0.92) and no interaction term (P = 0.84). The magnitude of the 24-hour response to LPS was then assessed by subtraction of the IL-10 concentrations found in the negative control cultures. The outcome of this calculation is shown in Figure 2A and provided, for each dietary group, a measure of LPS-stimulated IL-10 production above constitutive levels.

Figure 2A presents the outcome of a one-way ANOVA which revealed no diet-related effect on the production of IL-10 by splenic and nodal mononuclear cells in response to LPS *in vitro* (P = 0.47; pooled SEM = 36.1). Each bar represents a mean value (C: age-matched control group; LP: low-protein group; R: restricted-intake group) and is accompanied by its corresponding SEM.

#### 3.2.4. IL-10 production by mononuclear cells stimulated *in vitro* with anti-CD3

The concentration of IL-10 in culture fluids was expressed per 10^5^ CD3^+^ mononuclear cells, and analysis of these data by two-way ANOVA revealed an effect of stimulus (P = 0.0081) but no effect of diet (P = 0.16) and no interaction term (P = 0.70). With respect to the effect of stimulus, plate-bound anti-CD3 increased IL-10 production (when compared to PBS) but addition of anti-CD28 to provide a second stimulus made no difference to IL-10 concentrations relative to stimulation with anti-CD3, alone (Tukey’s Studentized Range test, P > 0.05). Therefore, the magnitude of the 24-hour response to anti-CD3 stimulation was determined by combining the outcomes of stimulated cultures independently of the involvement of the CD28 receptor followed by subtraction of the IL-10 concentrations found in corresponding negative control (PBS) cultures. The outcome of this calculation is shown in Figure 2B and provided, for each dietary group, a measure of anti-CD3-stimulated IL-10 production by T cells above constitutive levels.

As shown in Figure 2B, a one-way ANOVA revealed no diet-related difference in the production of IL-10 in response to anti-CD3 *in vitro* when expressed on a per 10^5^ splenic and nodal CD3^+^ cell basis (P = 0.32; pooled SEM = 0.18). In the Figure, bars represent mean values (anti-logs of means from natural log-transformed data) for each dietary group (C: age-matched control; LP: low-protein; R: restricted-intake). The data were pooled across stimuli (CD3 ± CD28) because the second stimulus, through the CD28 receptor, elicited no additional IL-10 production beyond the response generated through the CD3 receptor. An SEM is shown for each mean value.

The foregoing analysis and calculation required an assessment of the percentage of CD3^+^ (T) cells within the mononuclear populations recovered from the spleen and lymph nodes. As reported previously in the same experimental systems [17,18], the percentage of CD3^+^ mononuclear cells was higher in the low-protein group than in the age-matched controls (means: 44.7% and 29.9%, respectively), whereas the restricted-intake group (mean: 39.0%) did not differ from either of the other two dietary groups in this respect (Tukey’s Studentized Range test, pooled SEM = 3.36).

## 4. Discussion

The present investigation centered on challenge-induced production of IL-10, a component of second-tier anti-inflammatory immune regulation, in relevant and metabolically distinct forms of acute malnutrition in the weanling mouse. The challenge imposed in these investigations dictated the nature of the responses generated *i.e.*, an innate or adaptive immune response elicited by way of LPS or anti-CD3, respectively. The findings confirm a previous report [7] that constitutive IL-10 production *in vivo* is sustained even in the advanced stages of diverse forms of pre-pubescent malnutrition. By contrast, innate-type IL-10 production induced by challenge *in vivo* emerges as less robust than constitutive production of this anti-inflammatory cytokine independently of the metabolic form of acute malnutrition. Moreover, adaptive-type second-tier IL-10 production appears particularly fragile in the marasmic form of pathology. Malnutrition-associated loss of the second-tier component of anti-inflammatory competence, therefore, may provide an early sign that the ability to sustain regulated immune competence is beginning to fail. Perhaps of more fundamental significance, the influence of acute malnutrition on second-tier production of IL-10, at least of the adaptive type, appears dependent on the metabolic form of malnutrition pathology. This outcome could not be predicted by a model centered on chaotic disintegration of immunological capabilities, but is accommodated by the tolerance hypothesis of malnutrition-associated inflammatory decline. Notably, these significant conclusions were possible only pursuant to the studies conducted *in vivo*, and utilization of the novel technique of *in vivo* cytokine capture permitted a classic *in vitro* strategy for studying cytokine responses to be judged by the outcome of an *in vivo* procedure. As a result, and within the limits of a focused experimental study centered on a single cytokine, this investigation highlights the risk of extrapolating *in vitro* findings to the biological reality of intact organisms.

The timeframe of the response to LPS in this investigation, both *in vivo* and *in vitro*, precludes involvement of adaptive immunity, e.g., a thymic-independent humoral response [32]. Consequently, this component of the study centers on an innate-type response mediated through TLR-4 signaling [23]. The cellular compartment known to both express TLR-4 and produce IL-10 includes dendritic cells, mononuclear phagocytes, B cells and activated T cells [33–37], and any combination of these elements could have contributed to the outcome reported herein. In relation to the adaptive-type cytokine response, the effector/memory T cell compartment was isolated from its naïve precursor pool in this investigation, and involvement of newly-activated naïve T cells can be excluded for two independent reasons. First, to achieve effector status, naïve T cells require more than the short response periods studied herein [38,39]. Secondly, naïve T cells cannot differentiate to effector status if stimulated only through the T cell receptor [40]. However, in the present investigation, the *in vivo* response was elicited by stimulation exclusively through the CD3 molecule and, *in vitro*, co-stimulation through CD28 failed to increase the production of IL-10. The findings reported herein, therefore, separately reflect the IL-10-centered regulatory potential of the TLR-4-mediated innate defence compartment and of the adaptive effector/memory T cell compartment under challenge by external stimuli.

The *in vivo* cytokine capture assay and its interpretation are at the centre of this investigation. No *in vivo* index of cytokine production can be independent of physiological turnover, a potentially crippling confounder in relation to blood cytokines because of their brief half-lives that are generally measured in minutes [41–44]. The capture assay functions by trapping newly-synthesized cytokine in an antibody complex that extends the half-life of the bound cytokine to hours or days in the blood of the mouse [42,44–47]. This assay strategy is simply an extension of the physiological phenomenon whereby the clearance of cytokines from the blood is retarded when these molecules form complexes with soluble cytokine receptors [48] or anti-cytokine autoantibodies [49]. Therefore, when performed with a suitably short assessment period, e.g. four hours as in the present investigation, the capture assay is not overwhelmed by turnover and yields an index relating to net systemic cytokine production rate *in vivo*. The *in vitro* system used herein yields results that may relate more directly to cytokine production, at least on a per cell basis, than the capture assay. However, because of the non-physiological context of a cell culture microenvironment, the findings stemming from *in vitro* stimulation serve best in a supplementary role.

The metabolic dissimilarities between mice subjected to the low-protein and restricted-intake protocols, and their respective similarity to the human pathologies of kwashiorkor and marasmus, have been noted elsewhere [5,14–18]. Briefly, the low-protein protocol centers on a dietary imbalance, and weanling mice subjected to this protocol develop fatty liver and edema, the hallmark signs of kwashiorkor, after four weeks of wasting disease. When imposed for the periods of time reported in this investigation, therefore, the low-protein protocol produces an incipient kwashiorkor. Importantly, this protocol permits relative preservation of fat, *i.e.*, relative to the restricted-intake model, as would be anticipated in a model relevant to kwashiorkor. In contrast, the restricted-intake protocol induces weight loss apart from dietary imbalance. As expected in a model of marasmus, this protocol stimulates extreme mobilization of fat reserves but fails to produce the hepatic pathology or edema seen in mice subjected to the low-protein protocol. Despite their qualitative metabolic differences, these forms of experimental malnutrition have consistently failed to exhibit distinct immunological characteristics [1,5,16–18,28,29]. The findings of this investigation pertaining to innate-type second-tier production of IL-10 sustain this pattern, although it is noteworthy that a true decline did not occur in this index. Rather, its normal ontogenetic rise was attenuated. By contrast, a bona fide early-stage decline was apparent in second-tier IL-10 production by the effector/memory T cell compartment in the model of pre-pubescent marasmus, whereas no change in this immunological index was apparent even in the advanced stages of the model of incipient kwashiorkor. The significance and basis of this seemingly rare immunological distinction between forms of malnutrition pathology deserve research attention.

To the extent discernible from the *in vitro* studies of this investigation, acute weanling malnutrition imposes no fundamental influence either on the capacity of mononuclear cells to produce IL-10 in an innate-type response to external challenge or on the capacity of effector/memory T cells to produce this cytokine in an adaptive-type response. It remains possible that challenge-induced production of IL-10 declines *in vivo* on a per cell basis, e.g., as could be inferred from a report of modestly reduced expression of LPS receptor proteins by acutely protein-deficient mice [50]. However, lymphoid involution out of proportion with weight loss is a consistent feature of acute pre-pubescent malnutrition [1,2], and this phenomenon extends to the compartment of cells known to both express TLR-4 and produce IL-10. Therefore, loss of LPS-responsive elements appears sufficient to explain the inability to sustain a normal ontogenetic rise in innate-type challenge-evoked IL-10 production in the two forms of malnutrition pursued in this investigation. An involution-centered interpretation also appears sufficient with respect to the outcome of adaptive-type IL-10 production in the model of marasmus. In fact, within the depleted T cell system that is characteristic of acute malnutrition [1,2], loss of elements exhibiting an effector/memory phenotype is particularly profound in the murine models used herein [15,51,52]. In this light, the robust systemic effector/memory IL-10 response in the model of incipient kwashiorkor is all the more remarkable.

The tolerance model of malnutrition-associated immune depression proposes a purposeful anti-inflammatory immune regulation that is sustained, despite a limited supply of energy and substrates, with a view to reducing the risk of autoimmune reactions in catabolic pathologies [1,5,7]. This model is the antithesis of the classic paradigm of chaotic immunological disintegration, although all aspects of immune competence ultimately must succumb to disintegrative loss if wasting disease is permitted to progress indefinitely. In view of the robustness of constitutive IL-10 production in diverse forms of weanling malnutrition [7], confirmed herein, a decline in this cytokine response to external stimuli may provide an early sign that the ability to regulate immune competence is beginning to fail. More fundamentally, the central proposition of the tolerance model is that the primary physiological purpose of immune competence in acute pre-pubescent malnutrition is to provide protection against challenge from self antigens rather than against external challenges. Therefore, the present investigation points directly to the tolerance hypothesis with its finding that constitutive production of IL-10 is sustained in acute forms of malnutrition while production of this anti-inflammatory cytokine generally declines in response to external challenges. Moreover, the tolerance hypothesis accommodates particularly well the finding that the capacity to sustain second-tier T cell IL-10 production in acute malnutrition depends on the metabolic form of the pathology and is independent of nitrogen or energy decrement. In this context, it is intriguing that second-tier anti-inflammatory competence appears to be least fragile in a form of protein and energy deficit that must create a particularly high risk of autoimmune reactions through its emphasis on mobilization of lean tissue with its abundance of self antigens. The present investigation, therefore, provides several new findings that are accommodated more easily by the tolerance hypothesis than by a model of inflammatory decline centered on a physiologically trivial disintegration of inflammatory competence.

## Figures and Tables

**Table 1:** Response to LPS stimulation *in vivo*: Performance outcomes of weanling mice after 3-day or 14-day experimental protocols initiated at 19 days of age 1.

	Dietary Group 2			
Index	C	LP	R	SEM
**Day 3**				
Initial body weight (g/mouse)	8.5	8.4	8.6	0.10
Final body weight (g/mouse)	10.4 ^X^	7.2 ^Y^	7.4 ^Y^	0.10
Food intake (g/mouse **·** 3d) 3	7.8 ^X^	4.5 ^Y^	3.6 ^Y^	0.20
Food intake (g/g body weight **·** d)^3^	0.19 ^X^	0.13 ^Y^	0.09 ^Z^	0.001
Carcass dry matter (g/100g wet weight)	30.3 ^X^	28.3 ^Y^	27.9 ^Y^	0.30
Carcass lipid (g/100g wet weight)^4^	9.2 ^X^	5.9 ^Y^	3.4 ^Z^	0.03
**Day 14**				
Initial body weight (g/mouse)	8.3	8.5	8.6	0.08
Final body weight (g/mouse) 4	17.5 ^X^	6.1 ^Y^	6.0 ^Y^	0.02
Food intake (g/mouse **·** 14d) 5	60.6 ^X^	18.2 ^Y^	12.6 ^Z^	0.01
Food intake (g/g body weight **·** d)	0.20 ^X^	0.11 ^Y^	0.08 ^Z^	0.002
Carcass dry matter (g/100g wet weight)	32.1 ^X^	28.1 ^Y^	26.5 ^Z^	0.26
Carcass lipid (g/100g wet weight)	10.4 ^X^	4.5 ^Y^	2.7 ^Z^	0.34

1 Mean values. Within a row, values not sharing a superscript letter differ (P ≤ 0.05) according to Tukey’s Studentized Range test.

2 C: group that consumed complete diet ad libitum; LP: group that consumed low-protein diet ad libitum; R: group that consumed complete diet in restricted daily quantities.

3 From ANOVA of squared-transformed data. Mean values are square roots of squared means.

4 From ANOVA of natural log-transformed data. Mean values are antilogs of log means.

5 From ANOVA of inverse-transformed data. Mean values are the inverse of the transformed means.

**Table 2:** Response to anti-CD3 stimulation *in vivo*: Performance outcomes of weanling mice after 3-day and 14-day experimental protocols initiated at 19 days of age 1.

	Dietary Group 2			
Index	C	LP	R	SEM
**Day 3**				
Initial body weight (g/mouse)	8.4	8.5	8.7	0.09
Final body weight (g/mouse)	10.1 ^X^	7.4 ^Y^	7.5 ^Y^	0.12
Food intake (g/mouse **·** 3 d) 3	7.5 ^X^	4.9 ^Y^	3.5 ^Z^	0.04
Food intake (g/g body weight **·** d) 4	0.18 ^X^	0.13 ^Y^	0.09 ^Z^	0.24
Carcass dry matter (g/100g wet weight)^3^	30.4 ^X^	29.6 ^X^	27.4 ^Y^	0.01
Carcass lipid (g/100g wet weight)	8.0 ^X^	6.1 ^Y^	3.8 ^Z^	0.23
**Day 14**				
Initial body weight (g/mouse)	8.8	8.6	8.8	0.10
Final body weight (g/mouse) 3	18.5 ^X^	6.5 ^Y^	6.2 ^Y^	0.02
Food intake (g/mouse **·** 14 d) 3	65.6 ^X^	16.6 ^Y^	11.2 ^Z^	0.03
Food intake (g/g body weight **·** d) 3	0.27 ^X^	0.10 ^Y^	0.07 ^Z^	0.02
Carcass dry matter (g/100g wet weight) 3	32.8 ^X^	27.9 ^Y^	26.8 ^Y^	0.02
Carcass lipid (g/100g wet weight) 3	10.0 ^X^	4.1 ^Y^	2.0 ^Z^	0.04

1 Mean values. Within a row, values not sharing a superscript letter differ (P ≤ 0.05) according to Tukey’s Studentized Range test.

2 C: group that consumed complete diet ad libitum; LP: group that consumed low-protein diet ad libitum; R: group that consumed complete diet in restricted daily quantities.

3 From ANOVA of natural log-transformed data. Mean values are antilogs of log means.

4 From ANOVA of inverse-transformed data. Mean values are the inverse of the transformed means.
